# SARS-CoV2 infection in symptomatic patients: interest of serological tests and predictors of mortality: experience of DR Congo

**DOI:** 10.1186/s12879-021-07003-9

**Published:** 2022-01-04

**Authors:** Jean-Robert Makulo, Madone Ndona Mandina, Placide Kingebeni Mbala, Roger Dimosi Wumba, Pierre Zalagile Akilimali, Yannick Mayamba Nlandu, Jerome Ossam Odio, Ben Izizag Bepouka, Murielle Mashi Longokolo, Eric Kasongo Mukenge, Guyguy Kamwiziku, Jonathan Mutombo Muamba, Augustin Luzayadio Longo, Crispin Muanza Lufu, Hervé Letin Keke, Marcel Mambimbi Mbula, Hippolyte Nanituma Situakibanza, Ernest Kiswaya Sumaili, Jean-Marie Ntuma Kayembe

**Affiliations:** 1 Cliniques Universitaires de Kinshasa, Faculté de Médecine, Université de Kinshasa, Kinshasa, Democratic Republic of the Congo; 2 Secrétariat Technique du Comité Multisectoriel de la Riposte Contre la Covid-19, Kinshasa, Democratic Republic of the Congo; 3 Ecole de Santé Publique, Faculté de Médecine, Université de Kinshasa, Kinshasa, Democratic Republic of the Congo

**Keywords:** COVID-19, Symptomatic patients, Serological tests, RT-PCR, Concordance, Mortality

## Abstract

**Background:**

In symptomatic patients, the diagnostic approach of COVID-19 should be holistic. We aimed to evaluate the concordance between RT-PCR and serological tests (IgM/IgG), and identify the factors that best predict mortality (clinical stages or viral load).

**Methods:**

The study included 242 patients referred to the University hospital of Kinshasa for suspected COVID-19, dyspnea or ARDS between June 1st, 2020 and August 02, 2020. Both antibody-SARS-CoV2 IgM/IgG and RT-PCR method were performed on the day of admission to hospital. The clinical stages were established according to the COVID-19 WHO classification. The viral load was expressed by the CtN2 (cycle threshold value of the nucleoproteins) and the CtE (envelope) genes of SARS- CoV-2 detected using GeneXpert. Kappa test and Cox regression were used as appropriate.

**Results:**

The GeneXpert was positive in 74 patients (30.6%). Seventy two patients (29.8%) had positive IgM and 34 patients (14.0%) had positive IgG. The combination of RT-PCR and serological tests made it possible to treat 104 patients as having COVID-19, which represented an increase in cases of around 41% compared to the result based on GeneXpert alone. The comparison between the two tests has shown that 57 patients (23.5%) had discordant results. The Kappa coefficient was 0.451 (p < 0.001). We recorded 23 deaths (22.1%) among the COVID-19 patients vs 8 deaths (5.8%) among other patients. The severe-critical clinical stage increased the risk of mortality vs. mild-moderate stage (aHR: 26.8, p < 0.001). The values of CtE and CtN2 did not influence mortality significantly.

**Conclusion:**

In symptomatic patients, serological tests are a support which makes it possible to refer patients to the dedicated COVID-19 units and treat a greater number of COVID-19 patients. WHO Clinical classification seems to predict mortality better than SARS-Cov2 viral load.

## Background

The recently discovered SARS-CoV-2 in Wuhan, China is responsible for the current 2019 Coronavirus disease (COVID-19) pandemic which causes serious health and socio-economic consequences in the world. Based on some available epidemiological surveys, it has been established that the latency period of the disease varies between 1 and 14 days with an average of 5 days [1, 2]. Many infected people are asymptomatic; when the signs appear, patients often have fever, asthenia and a dry cough [3]. Severe patients usually present with dyspnea and/or hypoxemia 1 week after the onset of symptoms, and they can progress to acute respiratory distress syndrome (ARDS), septic shock and multiple organ dysfunctions [3]. It appears that from the second day after contact with the virus, SARS-Cov-2 nucleic acids can be detected in nasopharyngeal swabs using the “reverse transcriptase polymerase chain reaction” methods (RT-PCR) or by “next generation sequency” (NGS) [4]. Detection of the virus can also be in lower respiratory tract secretions, blood, feces and other samples [4, 5]. Virus-specific IgM and IgG become detectable approximately 3–5 days after the onset of the disease with a peak during the second and third week [4, 6]; IgM concentration gradually decreases during the fifth week until it becomes undetectable by the seventh week, while IgG persists beyond the seventh week and reaches a titer at least 4 times higher during convalescence compared to the acute phase [4, 7].

Among other issues related to the care of COVID-19 patients, the fluctuating and unpredictable nature of its course remains a major concern for clinicians. Indeed, an asymptomatic patient can quickly progress to a severe form of the disease and die within a few days; some patients have good clinical tolerance despite a high viral load [8–10]. An effort to classify patients according to their clinical signs, other organs failure and paraclinical data (results of medical biology and imaging examinations) has been made by the World Health Organization (WHO) [3], however the correlation between the COVID-19 clinical presentation, serological tests and the viral load results is not always so clear. The national “Technical Secretariat of the Multisectoral Committee Against the COVID-19” (TSMCAC) recommends that the diagnosis of COVID-19 must be made following a holistic approach integrating epidemiological data, symptoms, clinical signs and etiological evidence by RT-PCR [11]. The chest scanner can help in case of diagnosis.

Even if molecular diagnosis (RT-PCR) remains essential, its access is still very limited in a context where there is a chronic lack of reagents in sufficient quantity as in the case in the DR Congo. Moreover**,** in some situations, this RT-PCR examination may give false negative results [4]. Therefore, serological tests can support the diagnosis in the event that RT-PCR is negative or not available when the epidemiological, clinical and chest CT arguments are strong. The main objective of the study was to assess the concordance between the SARS-CoV2 RT-PCR and serological tests (IgM/IgG) results performed in symptomatic patients admitted to hospital with suspected COVID-19. The secondary objective was to compare the patients mortality curves according to the COVID-19 WHO clinical classification versus the viral load expressed by the CtN2 (cycle threshold value of the nucleoproteins) and the CtE (envelope) genes of SARS- CoV-2 detected using GeneXpert.

## Methods

The present cohort study included 242 patients referred to the University hospital of Kinshasa (UHK) for suspected COVID-19, dyspnea or ARDS between June 1st, 2020 and August 02, 2020. Both antibody-SARS-CoV-2 IgM/IgG and RT-PCR method were performed on the day of admission to hospital. The patients who died before the laboratory samples (RT-PCR and serological tests) were excluded from the study. Information related to demographic data, clinical characteristics, laboratory parameters and outcomes of patients, were collected prospectively. The suspected cases of COVID-19 (epidemiological and clinical data, chest scanner) and confirmed diagnoses of COVID-19 (RT-PCR) were defined according to the WHO (Table 1) and national TSMCAC guidelines [3, 11].

**Table 1:** The WHO clinical classification of the COVID-19 [3]

Mild case	Moderate	Severe	Critically severe
Mild clinical manifestations, none imaging performance	Fever, dyspnea, pneumonia performance on X-ray or CT	Meet any of the following: Respiratory distress, RR ≥ 30/min Oxygen saturation ≤ 93% PaO2/FiO2 ≤ 300 mmHg	Respiratory failure needs mechanical ventilation Shock Combination with other organ failure, patients need intensive care monitoring and treatment

*CT* computer tomography, *RR* respiratory rate, *PaO2* arterial oxygen pressure, *FiO2* inspired oxygen fraction

### SARS-CoV2 serological tests

We used the QuickZen® tests from the ZenTech® firm delivered by the National Institute for Biomedical Research (INRB). The kit is intended for the qualitative detection of IgM and IgG antibodies to SARS-CoV-2 in human serum. Sample collection and analysis were performed according to the protocol recommended by the manufacturer [12]. For this study, we took into account the result of the first serological test done during the patient’s admission to the hospital. According to the manufacturer, the sensitivity and specificity of the tests are 97% and 99% for IgM vs 100% and 97% for IgG. After 12 days of positive RT-PCR result, 100% of patients have positive IgM and IgG [12].

### SARS-CoV2 RT-PCR test

The SARS-CoV2 RT-PCR test was performed by the GeneXpert method using a Cepheid® brand device and Cepheid brand SARS-Cov-2® cassettes [13]. In clinical samples, Xpert Xpress SARS-CoV-2 reaches an agreement of 100% compared to real time RT-PCRs [14]. Using sterile dacron rods, a sample was taken from the patient’s nostrils and another from the back of the throat by medical biology personnel previously trained in the INRB. The swabs thus collected were placed in a transport medium for the virus (MTV), which was stored in a cold chain. The sample was taken to the laboratory for analysis, which was carried out in a type 2 microbiological safety hood. Using a pipette, 1 ml of MTV was taken and deposited in the GeneXpert cassette. Then the cassette was placed in the machine (procedure according to the manufacturer, extraction-hybridization-amplification). The scan was completed after about 50 min. The results are expressed in the form of curves as a function of the Ct values of the N2 gene and of the E gene as well as that of the device control (SPC = sample process control). A result is said to be positive when the Ct value of the N2 and/or E genes are ≤ 40; a Ct value > 40 indicates a negative result [15].

### Operational definitions

Suspected case of COVID-19: any patient presenting clinical signs and/or visible signs on chest CT suggestive of COVID-19.

Confirmed case: any symptomatic patient meeting the laboratory criteria (RT-PCR and/or IgM or IgG positive).

### Statistical analyzes

Statistical analyses were performed using SPSS 21.0 for Windows (SPSS Inc., Chicago, IL, USA). Comparisons between the groups of patients were performed using Student’s t test, Fisher’s exact test and the Chi square test, when appropriated. The agreement between the two tests (RT-PCR and serological tests) was evaluated using the Kappa coefficient of Cohen. The Kaplan Meier curves were built for survival analyses. The Cox regression analysis was used to identify the independent predictors of mortality. Association measures were calculated with a confidence interval of 95%. In the survival analysis, the outcome was tested related to time since the beginning of COVID-19 symptoms until death. Survival was assessed from the first day of symptoms through day 42 (6 weeks). The time to consult, age, clinical stage of COVID-19 and the viral load (expressed by the CtN2 and CtE values) were the variables introduced in the Cox model. In the analyses, p-values below 5% (p-value < 0.05) were considered statistically significant.

### Ethical considerations

The rules of ethics and confidentiality were respected. All COVID-19 patients and other patients followed in the center received free medical care in accordance with the available therapy protocols. Our research projects on COVID-19 had been authorized by the School of Public Health Ethics committee, University of Kinshasa (N°ESP/CE/132/2020). Written informed consent was not obtained and was waived by the School of Public Health Ethics committee, University of Kinshasa for patient owing to the urgency and unprecedented nature of COVID-19 pandemic and the non-interventional nature of the study.

## Results

### General characteristics of patients

A total of 242 patients (65.7%) were included in the study. They had an average age of 51.5 ± 18.6 years with extremes of 3 and 86 years. Only 15 patients (6.2%) were under 18 years old while 137 patients (56.6%) were between 18 and 60 years old, and 90 patients (37.2) were over 60 years old.

The time between the first symptoms and the consultation was 7.4 ± 3.3 years (extremes: 2 and 23 days). The clinical presentations of the cases were mild, moderate, severe and critical in respectively 53.7%, 13.2%, 19.8% and 4.1%. Table 2 which compares the confirmed cases of COVID-19 (104 patients) and the other patients, shows a difference with respect to age, clinical presentation and consultation time.

**Table 2:** Clinical presentation of suspected COVID-19 patients

	Whole group	Confirmed case	Not-confirmed	p
Men, n (%)	159 (65.7)	71 (68.3)	88 (63.8)	0.277
Age, years ± SD	51.5 ± 18.6	57.3 ± 16.5	47.2 ± 18.9	< 0.001
Age < 18 years, n (%)	15 (6.2)	4 (3.8)	11(7.8)	0.001
Age, 18–60 years, n (%)	137 (56.7)	48 (46.2)	89 (64.5)	
Age ≥ 60 years, n (%)	90 (37.2)	52 (50.0)	38 (27.5)	
Time before consultation, days	7.4 ± 3.3	8.3 ± 3.7	6.7 ± 2.8	< 0.001
Mild case, n (%)	130 (53.7)	33 (31.7)	97 (70.3)	< 0.001
Moderate case, n (%)	32 (13.2)	16 (15.4)	16 (11.6)	
Severe case, n (%)	48 (19.8)	36 (34.6)	12 (8.7)	
Critical case, n (%)	10 (4.1)	8 (7.7)	2 (1.4)	

### RT-PCR and serological tests results

The RT-PCR was positive in 74 patients (30.6%). Seventy two patients (29.8%) had positive IgM and only 34 patients (14.0%) had positive IgG. The combination of RT-PCR and serological tests made it possible to treat 104 patients as having COVID-19, which represented an increase in cases of around 41% compared to the result based on RT-PCR alone.

The comparison between RT-PCR and serological tests results has shown that 47 patients were declared positive on both tests and 138 patients were negative on both tests (Table 3). In total 57 (23.5%) patients had discordant results. The Kappa coefficient of agreement between the two tests was 0.451 (p < 0.001).

**Table 3:** Agreement between RT-PCR and serological tests

	RT-PCR positive n = 74	RT-PCR negative n = 186	p
IgM + or IgG +	47	30	< 0.001
IgM − and IgG −	27	138	
IgM + and IgG +	17	13	0.002
IgM + and IgG −	28	14	< 0.001
IgM − and IgG +	2	3	0.643
IgM +	46	26	< 0.001
IgG +	19	15	0.001

### Mortality according to clinical classification and viral load expressed by Ct values

**Fig. 1:**
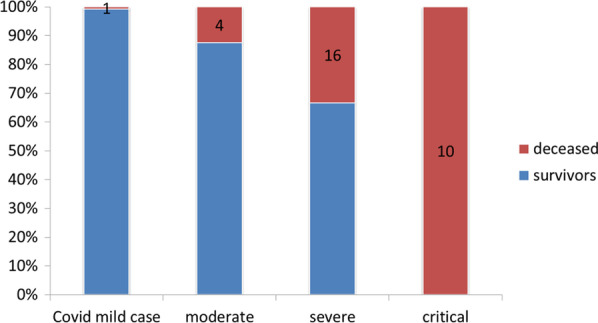
Survival according to WHO COVID-19 clinical classification

**Fig. 2:**
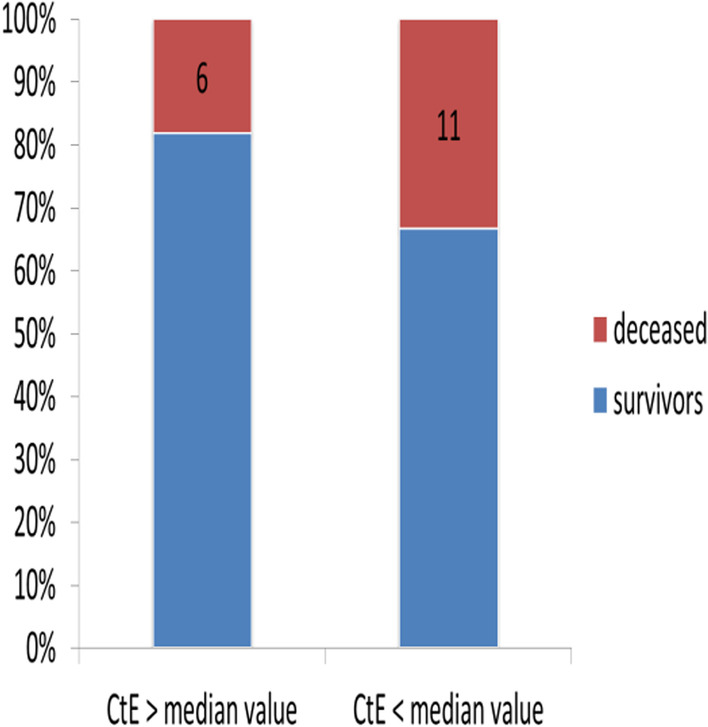
Survival according to viral load expressed by CtE

**Fig. 3:**
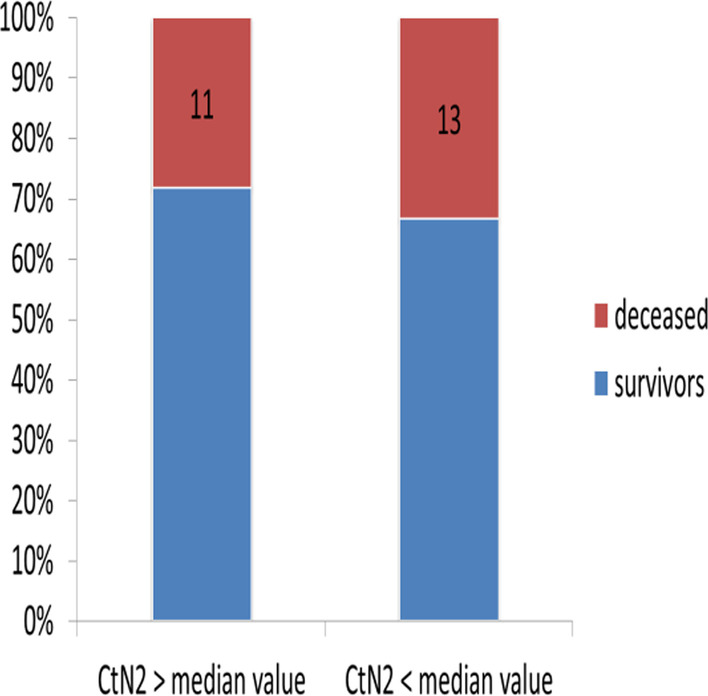
Survival according to viral load expressed by CtN2

We recorded 23 deaths (22.1%) among the 104 patients treated as having COVID-19. In the group without COVID-19, 8 deaths (5.8%) were recorded among the 138 patients followed. Figures 1, 2, 3 show the survival rates of the patients according to the stages of the disease (WHO clinical classification) and the viral load expressed by the values of CtN2 and CtE. The patients who survived represented 97.0%, 87.5%, 66.7% and 0% depending on whether the disease was classified as mild, moderate, severe and critical (p < 001). All critically ill patients had died, their median survival from the onset of symptoms was 8 days with a 95% CI between 5.783 and 10.217 days.

**Fig. 4:**
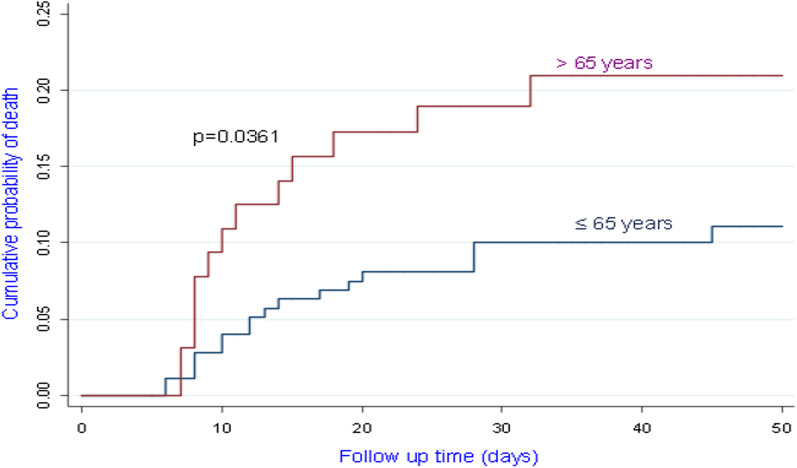
Survival curves according to patient age

**Fig. 5:**
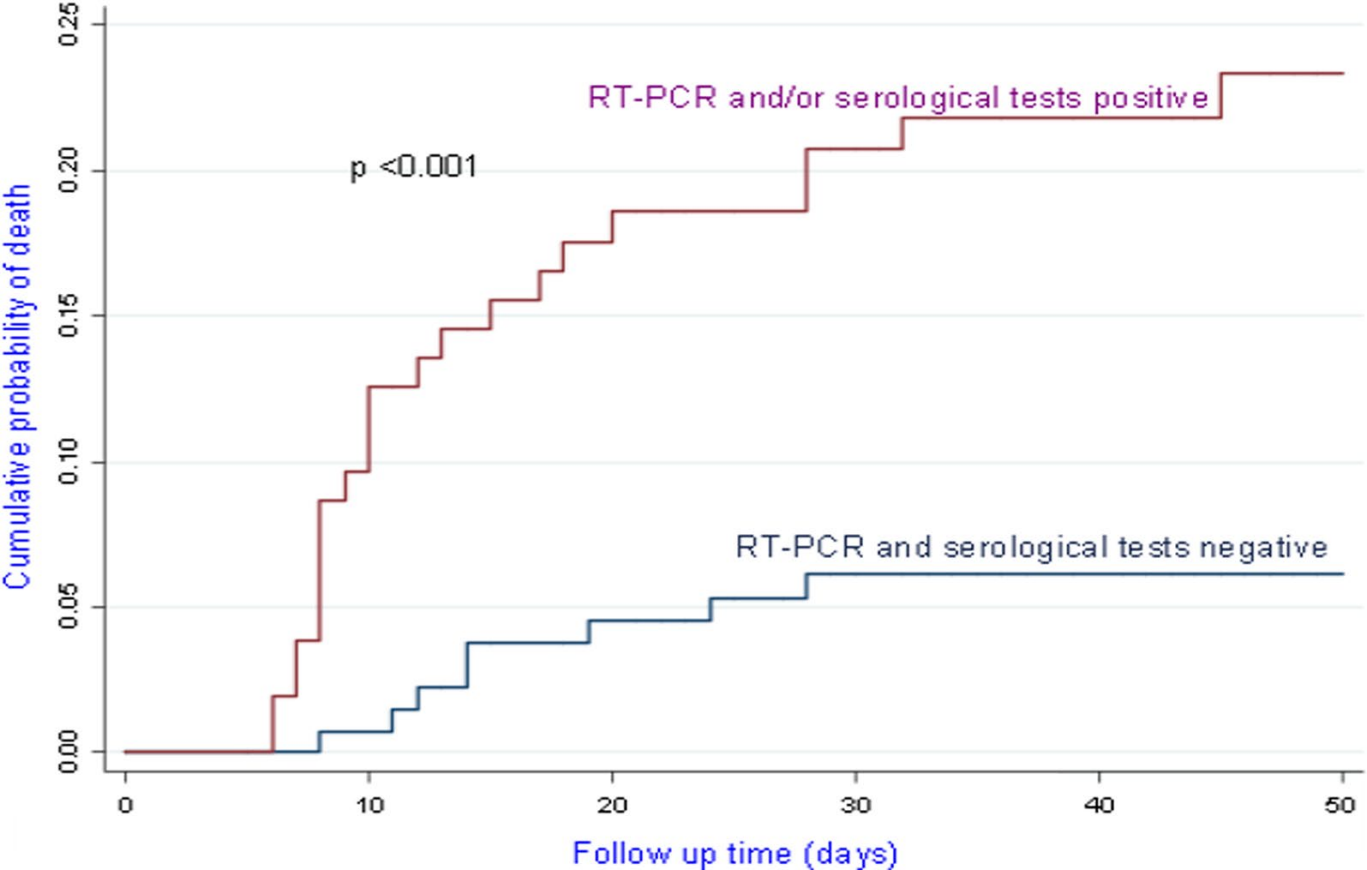
Survival curves of COVID-19 vs non COVID-19 patients

**Fig. 6:**
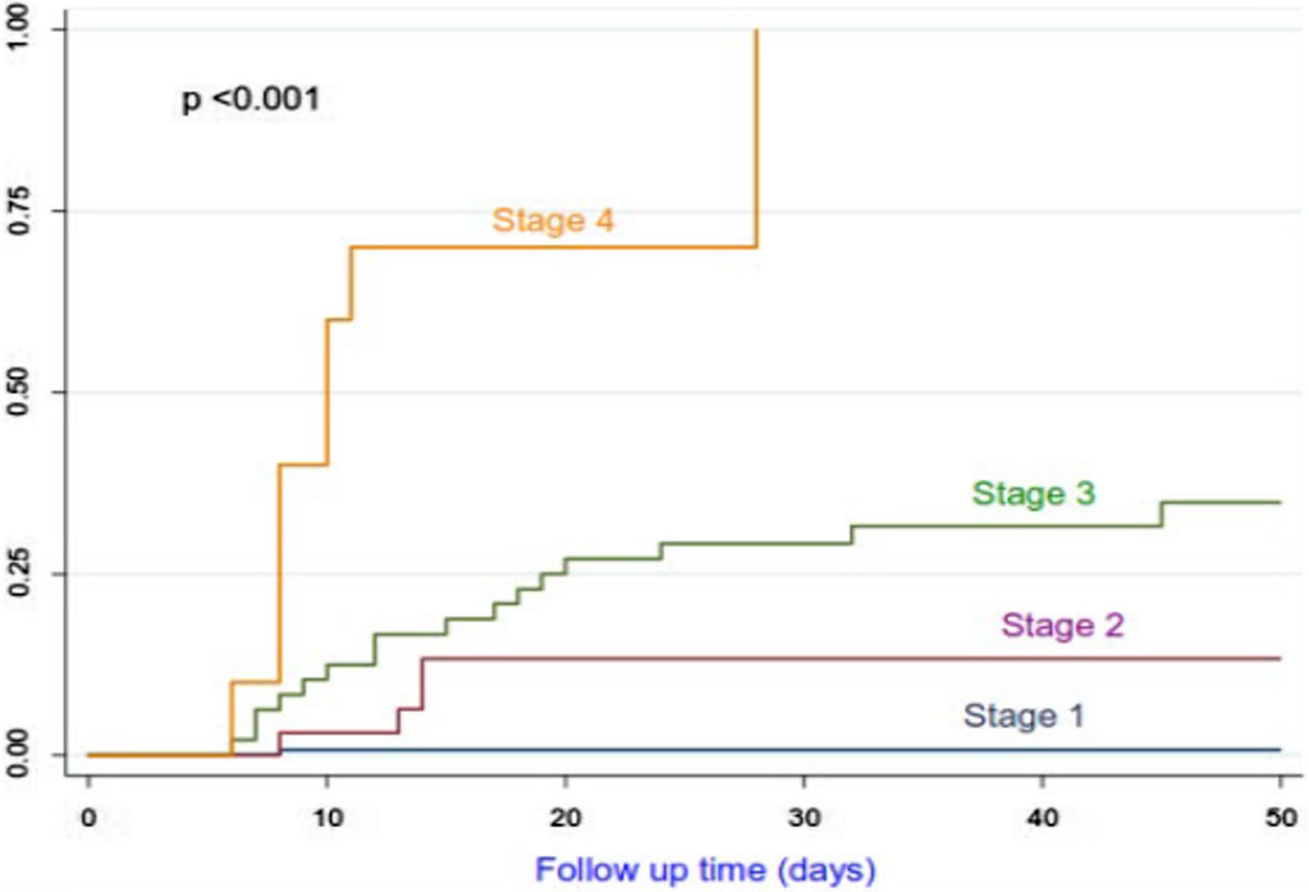
Survival curves of COVID-19 patients according to the clinical stage of the disease

**Fig. 7:**
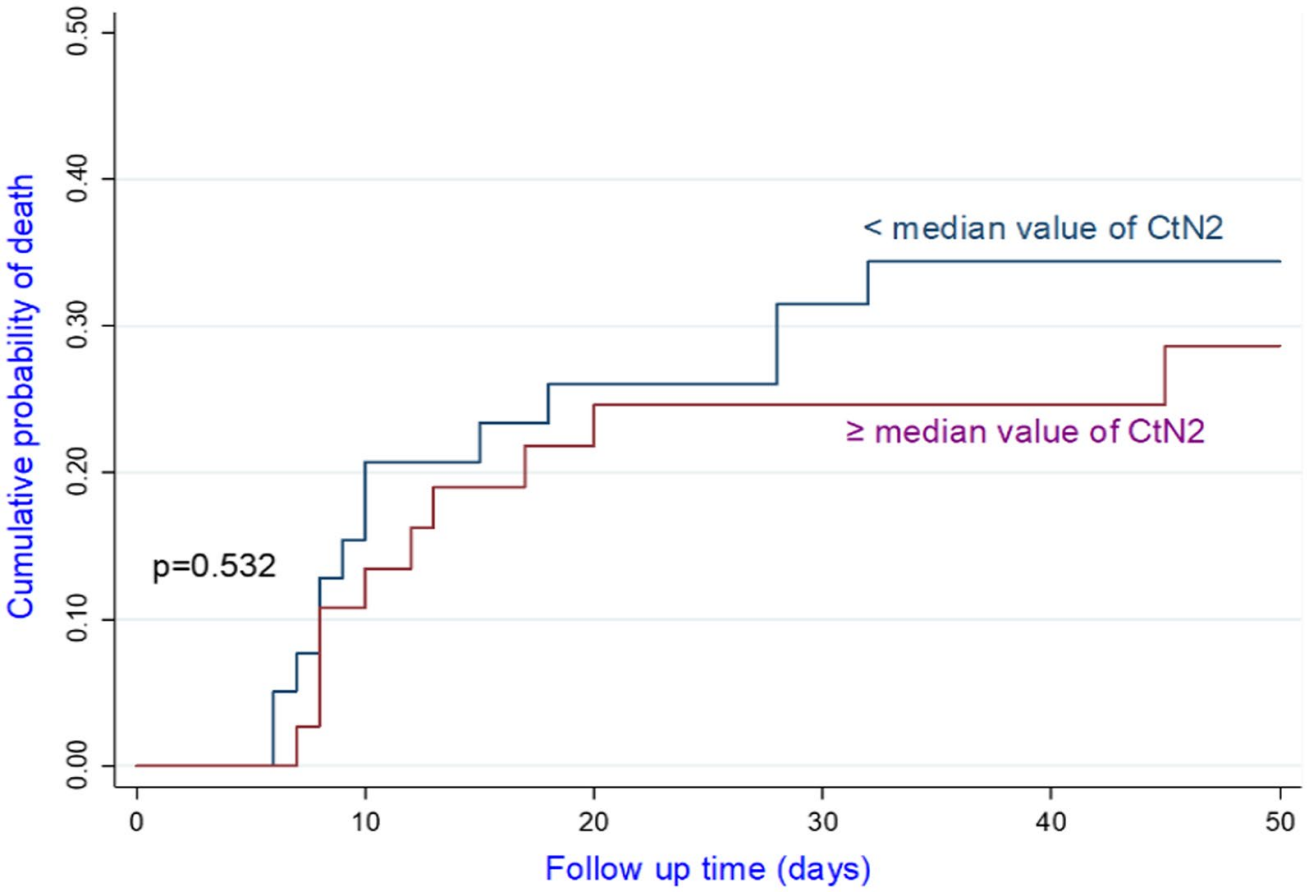
Survival curves as a function of the CtN2 value

**Fig. 8:**
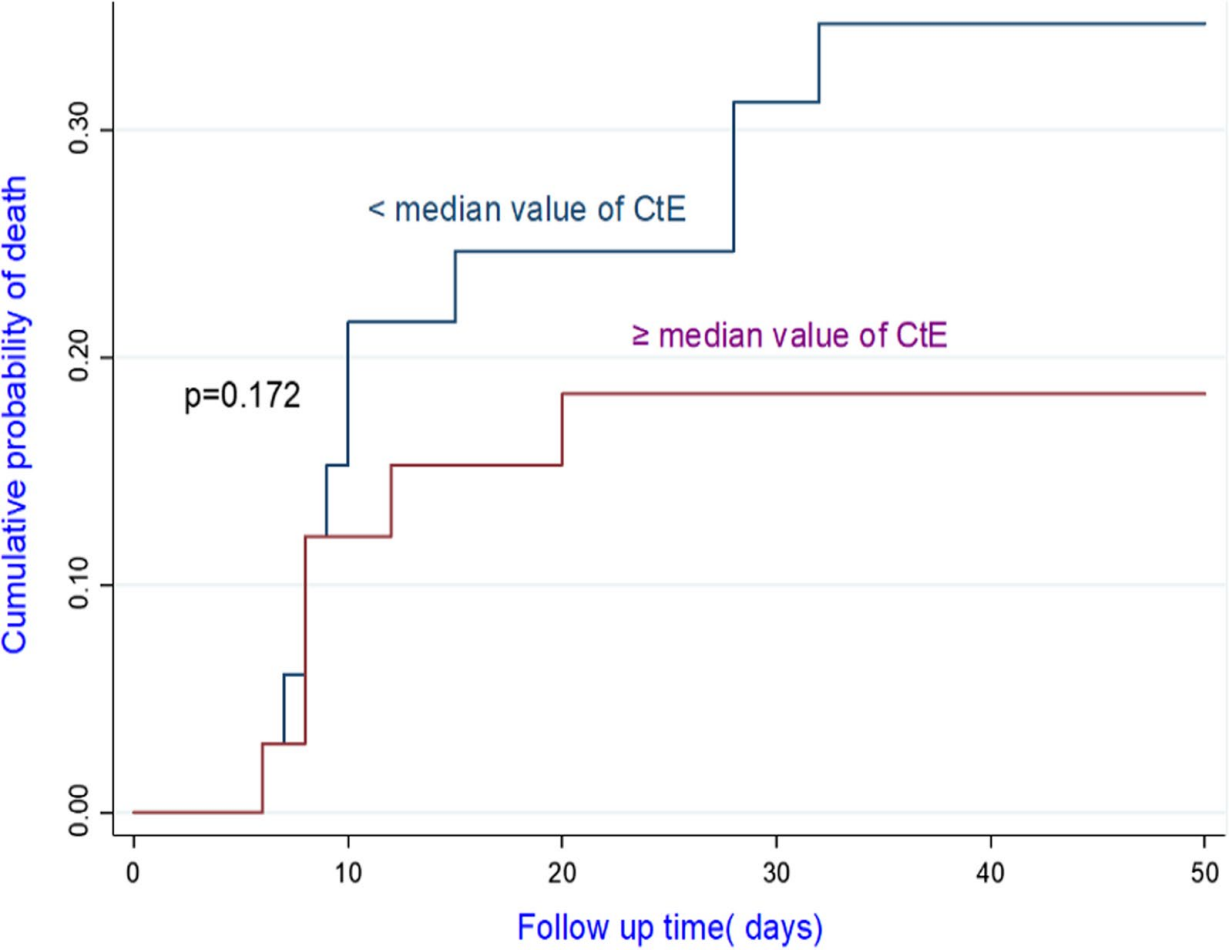
Survival curves as a function of the CtE value

Among the confirmed case, 81.8% of patients with CtE above the median value survived vs 66.6% of patients who had CtE below the median value (p = 0.130); 71.8% of patients with CtN2 above the median value survived vs 66.6% of patients who had CtN2 below the median value (p = 0.403). The Kaplan-Meir curves showed a better survival in younger patients and in patients who did not have the COVID-19 (Figs. 4 and 5). Among COVID-19 patients, mortality increased proportionally with the clinical stage of the disease (Fig. 6). Although having better survival compared to the other group, patients with a lower viral load did not have a statistically significant advantage (Figs. 7 and 8). In Cox analysis, the predictor (covariate) variables which were assumed to influence the survival of patients included in the model were (1) age, (2) IgG, (3) viral load expressed by the CtE and Ct values (4) clinical stage of COVID-19 and (5) time between the first symptoms and the hospital visit. Analyzes revealed that only COVID-19 clinical stage and time between first symptoms admission to hospital were independent predictors of survival (Table 4).

**Table 4:** Predictors of death during the study period

Covariates	n	Crude HR	p	aHR	CI 95%	p
Time before consultation	242	1.000	0995	0.868	0.769–0.969	0.021
Severe-critical cases	184	19.787	< 0.001	26.795	10.027–71.599	< 0.001
Confirmed case of COVID-19	104	4.136	0.001	–	–	–
Age > 65 years	65	2.101	0.041	–	–	–

*HR* Hazard ratio, *aHR* adjusted Hazard ratio

## Discussion

This prospective cohort conducted in symptomatic and suspected patients of COVID-19 has shown that by combining serological and molecular tests, we are able to treat a larger number of patients by considering them to have COVID-19; moreover, the risk of dying is better predicted by using the clinical classification in relation to the viral load of SARS-CoV2.

The study period coincides with the peak of the COVID-19 pandemic in the city of Kinshasa. Hospitals experienced a significant flow of patients and several cases of death were reported. COVID-19 Patients were hospitalized in isolated units with dedicated staff and the management of ARDS was initially limited to a few hospitals. Hospitals have been equipped (noninvasive ventilation, Optiflow, Respirators) gradually but the number of equipment remains limited in case there was a real outbreak of disease. In a complex scenario such as the ongoing pandemic, not only the diagnoses need to be timely and accurate, but laboratory testing needs also to provide information to avoid missing cases. By combining RT-PCR and serological tests, 43% of patients who had symptoms and or chest CT suggestive of COVID-19 were treated. With RT-PCR alone, we were going to limit ourselves to 31%. Although being a gold standard method for confirming the SARS-coV2 infection, the limitations of RT-PCR, both real-time and GeneXpert, are known. False-negative results have been reported at rates as high as 20–40% in cases for which both clinical symptoms and imaging evidence raised strong suspicions of disease [4, 16]. False negatives may be caused by various factors, including the specimen source, timing of sampling, personnel operation, and the test kit quality [4, 16]. Instead of waiting for a second RT-PCR result as some recommend (in the event of a first negative test) [17, 18], we believe that this strategy wastes the time to diagnose the disease (1–2 days or more), with a very high cost. Even considering the serological window which sometimes limits the value of serological tests, in our situation where patients consult the hospital several days after the onset of symptoms, serological assays can become helpful both to complete the epidemiological link when molecular diagnosis results are negative, and to alleviate the burden of laboratories implicated in molecular diagnosis. The time for seroconversion is about 10–14 days, but early seroconversion has also been documented at 3–5 days post infection [4–7]. Table 2 shows that patients in whom the diagnosis of COVID-19 was confirmed were older, consulted later in the hospital and had a more serious clinical picture. The stigmatization of patients, the denial of the disease by a large part of the population and the habit of consulting late in the hospital after self-medication largely explain these results.

Contrary to the COVID-19 WHO clinical classification, the viral load result (expressed by CtE and CtN2 values) had no prognostic value to predict the mortality. Pujadas et al. had showed an independent relationship between high viral load and mortality; so transforming qualitative testing into a quantitative measurement of viral load could assist clinicians in risk-stratifying patients and choosing among available therapies and trials [19]. Our study may have the disadvantage of being monocentric with a small sample size. To date, the study of the viral load to predict the prognosis of COVID-19 has not been the subject of several publications. Cohort studies with large samples may resolve the issue. Nevertheless, independent of the viral load, several factors, in particular co-morbidities, explain the death in COVID-19 patients. In a recent systematic review and meta-analysis, Lu et al. had showed that advanced age as well as comorbidities and laboratory indicators including lactate dehydrogenase, C-reactive protein, neutrophil, Blood urea nitrogen and albumin are correlated with COVID-19 mortality [20].

Among the risk factors studied, the clinical stages of the disease (severe and critical COVID-19) as well as the time between the first symptoms and admission to hospital were retained in the Cox model. Even if the risk linked to the stage of the disease can be explained, the probability of dying, which is multiplied by 26, shows how the management of severe forms of COVID-19 remains a major concern. In countries with low resources and where the health system is fragile, such as the DR Congo, efforts must focus on preventive measures that require compliance with barrier measures. The shorter hospital admission time for deceased patients reflects the fact that in our environment, individuals come to the hospital when the situation is serious. Likewise, it is possible that the lethal strains of the virus also have a shorter latency time; which may explain a faster hospital consultation. A limitation that must be recognized is the fact that in severe patients, the information was obtained by hetero-history. The exact date of onset of symptoms may be wrong. Although the patients who died were relatively older, this did not emerge as an independent risk factor in statistical analyzes. We have already mentioned the limit related to the size of the sample.

## Conclusion

Considering that patients come to the hospital late, serological tests are a support which makes it possible to treat a greater number of patients who present the clinical signs/CT scan of COVID-19, especially when RT- PCR is negative. The SARS-CoV2 viral load expressed by the values of CtE and CtN2 does not appear to have prognostic value in predicting death. Multicenter cohort studies and meta-analyzes must be carried out to have definitive conclusions.

## Data Availability

The datasets used and/or analysed during the current study are available from the corresponding author on reasonable request.

## Abbreviations

ARDS: Acute respiratory distress syndrome
CT: Computer tomography
COVID-19: Coronavirus disease 2019
CtE: Cycle threshold value of the envelope
CtN2: Cycle threshold value of the nucleoproteins
FiO2: Inspired oxygen fraction
INRB: National Institute for Biomedical Research
MTV: Transport medium for the virus
NGS: Next generation sequency
PaO2: Arterial oxygen pressure
RR: Respiratory rate
RT-PCR: Reverse transcriptase polymerase chain reaction
SARS-Cov-2: 2019 Novel coronavirus
TSMCAC: Technical Secretariat of the Multisectoral Committee Against the COVID-19
UHK: University hospital of Kinshasa
WHO: World Health Organization
